# The potential protective effects of temporal bone pneumatization: A shock absorber in temporal bone fracture

**DOI:** 10.1371/journal.pone.0217682

**Published:** 2019-05-31

**Authors:** Tae Kyu Kang, Ryun Ha, Jae Hwan Oh, Woongsang Sunwoo

**Affiliations:** Department of Otorhinolaryngology-Head and Neck Surgery, Gachon University Gil Medical Center, Incheon, Korea; Duke University School of Medicine, UNITED STATES

## Abstract

**Objectives:**

We hypothesize that when temporal bone fractures occur, the pneumatic cells in the temporal bone are able to absorb most of the impact force during a traumatic event. This study aims to correlate the degree of pneumatization of the temporal bone with the severity of temporal bone fracture (TBF).

**Methods:**

Charts and computed tomography scans representing 54 TBFs, diagnosed from 2012 to 2017 at a single tertiary hospital, were retrospectively reviewed. Temporal bone pneumatization (TBP) in the petrous apex and mastoid region was evaluated using previously published classification systems. TBP classifications and fracture types were correlated with TBF complications such as sensorineural hearing loss (SNHL), facial nerve palsy (FNP), and vestibular dysfunction.

**Results:**

Patients with increased pneumatization of the temporal bone had significantly fewer and less severe SNHL. SNHL more strongly correlated with the degree of pneumatization in the mastoid (P = 0.005) than that in the petrous apex (P = 0.024). On the other hand, the degree of TBP correlated poorly with FNP and vestibular dysfunction. However, the mastoid hypopneumatization demonstrated significant correlation with otic-capsule violations (P = 0.002). Fractures with otic-capsule violation were 4 times more likely to have vestibular dysfunction (P = 0.043) and 3 times more likely to have SNHL (P = 0.006). FNP was not associated with otic-capsule violating fractures but was 3.5 times more common in comminuted fractures (P = 0.025).

**Conclusions:**

The degree of temporal bone pneumatization was negatively correlated to the incidence of otic-capsule violation and the severity of hearing impairment in patients with temporal bone fracture. This study substantiated the potential protective effect of temporal bone pneumatization in TBFs.

## Introduction

In humans, temporal bone pneumatization (TBP) begins during prenatal development [1]. Pneumatization refers to both the process by which the epithelium expands into developing bone and the resulting interconnected air cells within the temporal bone [2]. Only humans and primates have pneumatized spaces such as the mastoid air-cell system in temporal bones [3]. Studies suggested that the extent of pneumatization has decreased over the course of human evolution and usually limited to the mastoid, the petrous apex, and the perilabyrinthine regions in human temporal bones [4]. However, little is known about the evolutionary value and/or functional significance of these pneumatized spaces. While the function of TBP remains unknown, numerous previous studies reported the correlations between the degree of TBP and various pathologies including atelectasis, otitis media, and cholesteatoma [5–9]. Generally, poor pneumatization in temporal bone is closely associated with increased incidence and poor prognosis of these pathologic conditions. To explain this relationship, people have long debated the function of TBP and a number of theories have been proposed: 1) pressure buffer, 2) gas reserve, and 3) shock absorption spaces.

The tympanum and communicating air-cell system constitute one gas pocket and share the same pressure. For normal tympanum function as the peripheral transducer organ for hearing, intratympanic gas pressure needs to be maintained at atmospheric level [10]. Failure of maintaining pressure homeostasis may result in sustained negative pressure that may cause tympanic membrane retraction (atelectasis and retraction pocket cholesteatoma) or fluid accumulation. Well-pneumatized spaces in temporal bone greatly increase the volume of the tympanic cavity and may play a role of a pressure buffer [11]. By Boyle’s law, stating that pressure is inversely proportional to the volume ($P\propto \frac{1}{V}$), large air volume of TBP dilutes pressure changes and makes it relatively resistant to the associated effects.

Other hypothetical is that TBP function as gas reserve [12]. Under physiologic conditions, the sum of blood gas partial pressures is less than that of the middle ear by approximately 50 mm Hg [13]. In response to this gradient, intratympanic pressure tend to decrease by diffusive transmucosal gas exchange. According to the general gas law for a fixed volume system, large volume of TBP lessen the rate of pressure change caused by transmucosal loss of gas.

Because it is not clear whether reduced volume of TBP is predisposing factor of middle ear diseases or their result during childhood, the current study focused on shock absorption theory. We hypothesize that when temporal bone fractures occur, the pneumatic cells in the temporal bone are able to absorb most of the impact force during a traumatic event. The pneumatization may provide a survival benefit serving to protect surrounding vital organs, such as the cochlea, vestibule, facial nerves, and carotid artery. Previously, human cadaver studies have been performed in an attempt to substantiate this presumption [14, 15]. They showed that mastoid obliteration increased the severity of fracture compared to air spaces in preserved mastoids. However, limited data from the small number of samples did not provide information between the degree of TBP and the severity of temporal bone fractures. Thus, this study aims to correlate the degree of TBP, specifically the petrous apex and mastoid, with clinical sequelae associated with temporal bone fracture.

## Materials and methods

This retrospective study was approved by the institutional review board of the Clinical Research Institute at Gachon University Gil Medical Center (No. GFIRB2019-065). All methods employed in this study were in accordance with the approved guidelines and the Declaration of Helsinki. Data were collected from an electronic medical records database and analyzed anonymously. All clinical data were retrospectively reviewed by two otologists.

### Subjects

The electronic database of our trauma center containing information for all patients evaluated over the 6-year period from January 2012 to December 2017 was retrospectively reviewed. Temporal bone fracture was identified by Korean standard classification of diseases (KCD)-6 and KCD-7 code S02.18 (“other fracture of base of skull”), which is the same code as S02.19 (“other fracture of base of skull”) in the 2019 edition of international classification of diseases, tenth revision, clinical modification (ICD-10-CM). This study population included only patients who underwent temporal bone computed tomography (TBCT) within 1 month after head trauma and had confirmatory diagnosis of temporal bone fracture by radiologists. Basic demographics, the mechanism of injury, and clinical sequalae of the fracture were examined based on medical records. The clinical sequalae evaluated included hearing loss, facial nerve paresis or paralysis, and vestibular disorders. Sensorineural hearing loss (SNHL), including the sensorineural component of the mixed hearing loss, was confirmed objectively by pure tone audiometry. The diagnosis of vestibular dysfunction and benign paroxysmal positional vertigo (BPPV) was made on vestibular function tests (VFTs), including videonystagmography (VNG) and bithermal caloric test. Caloric examinations were not performed on all patients. Exclusion criteria included the absence of an audiogram, incomplete or unclear medical records, isolated fractures of the squamous portion of the temporal bone, and isolated tympanic plate fractures associated with mandibular injuries.

### Radiologic evaluation

A total of 49 high-resolution TBCT scans reporting a temporal bone fracture were identified which met our selection criteria for evaluation. Two otologists blinded to the clinical findings separately evaluated the temporal bone fractures in both axial and reconstructed coronal planes and made final decisions by consensus.

Each temporal bone fracture was classified as otic-capsule violating (OCV) or otic-capsule sparing (OCS) and transverse, longitudinal, or oblique according to the traditional classification systems. The otic capsule is the rigid bony outer wall of the inner ear in the temporal bone, which is the most dense portion of the temporal bone on radiographic images. The involvement of otic-capsule was defined by the presence of fracture lines on at least 1 of the walls surrounding the otic capsule, regardless of the fracture plane. Transverse fracture was defined as fracture running perpendicular to the petrous ridge. Longitudinal fracture was defined as fracture coursing parallel to the petrous ridge. Oblique fracture was defined as fracture crossing the petrotympanic fissure and running oblique planes on the external surface of the temporal bone.

**Fig 1:**
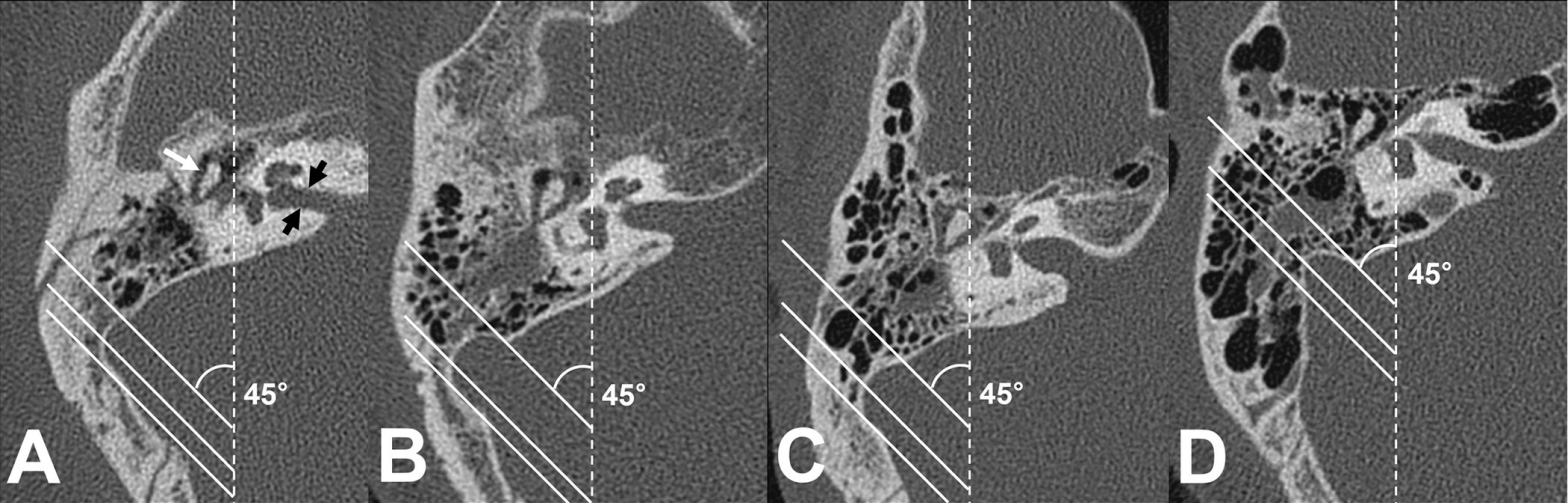
Degrees of mastoid pneumatization with reference to the sigmoid sinus. At the axial section where the malleoincudal complex (white arrow) appeared as an ice cream cone shape and the internal acoustic canal (black arrows) was identified clearly, three parallel lines angled at 45° to the anteroposterior axis (dotted line) were drawn. (A) Poor pneumatization: pneumatization remains anteromedial to the anterior line (passing the most anterior point). (B) Moderate pneumatization: pneumatization is between the anterior and middle (passing the most lateral point) lines. (C) Good pneumatization: pneumatization is between the middle and posterior (passing the most posterior point) lines. (D) Very good pneumatization: pneumatization is extended posterolaterally beyond the posterior line.

The mastoid pneumatization was evaluated using the classification system proposed by Han et al [16]. The axial section in which the ice cream cone shaped malleoincudal complex, formed by the joint of ossicles between the malleus head and the incus body, and the internal acoustic canal were identified most clearly was selected as the standard section. Three parallel lines were drawn in relation to the sigmoid sinus. These lines are placed in 45° angle to the anteroposterior axis of the image. The anterior, middle and posterior lines crossed the most anterior point, the most lateral aspect, and the most posterior point of the sigmoid sinus, respectively. Temporal bone pneumatization was classified into four groups: Poor pneumatization, the pneumatization remains anteromedial to the anterior line; Moderate pneumatization, the pneumatization extending between the anterior and middle lines; Good pneumatization, pneumatization between the middle and posterior lines; Very good pneumatization, pneumatization extending posterolaterally beyond the posterior line (Fig 1). The degree of mastoid pneumatization differed between interpreters in 5.6% (3/54) of temporal bones. In those cases, two blinded interpreters, who were not aware of their previous interpretations, reperformed the assessments together with caution. Then, the final decisions were made without controversy.

**Fig 2:**
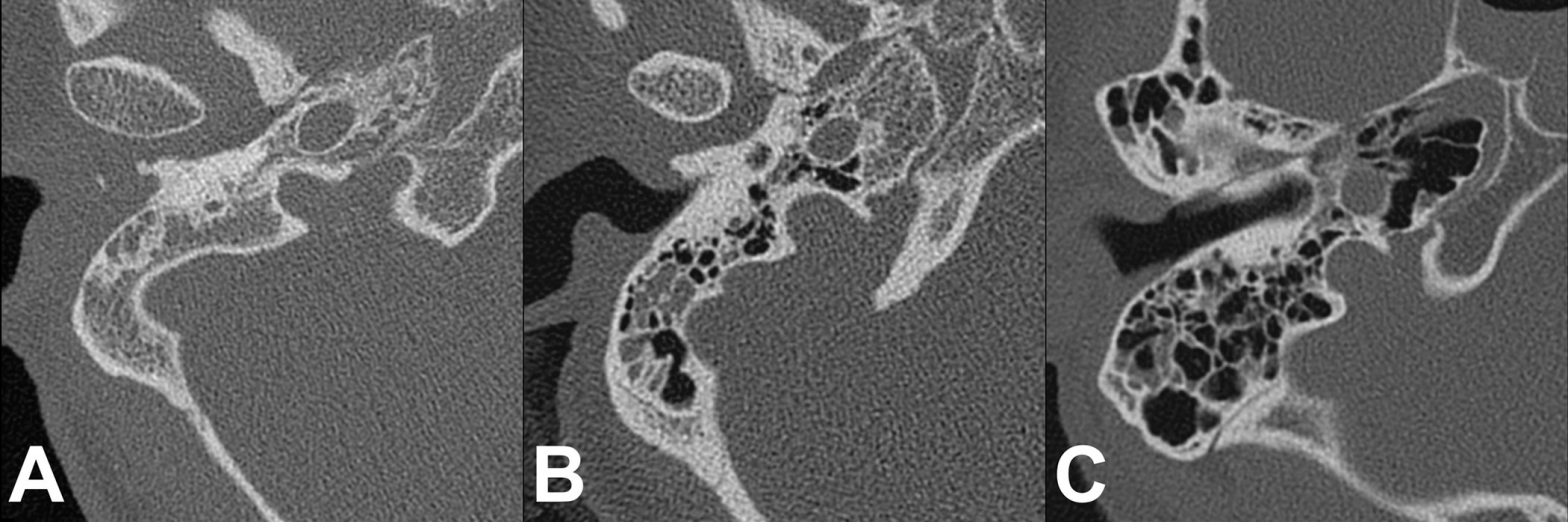
Degrees of petrous apex pneumatization with reference to the carotid canal. (A) None: no pneumatization present in the petrous apex. (B) Mild pneumatization: focal pneumatization is found either medial or lateral to the carotid canal. (C) Complete pneumatization: pneumatization is surrounding the carotid canal.

The petrous apex pneumatization was classified into three groups according to the study conducted by Jadhav et al [17]. The ascending carotid artery on axial plane was used as a reference structure: None, no evidence of pneumatization in the petrous apex; Mild pneumatization, mild pneumatization either medial or lateral to the carotid canal; Complete pneumatization, complete pneumatization surrounding the carotid canal (Fig 2).

### Audiometric evaluation

The pure tone audiometry data of all subjects were evaluated. The procedure for pure-tone audiometry was based on an international standard (ISO 8253) and American national standards institute (ANSI) standards. Testing was performed in a double walled sound treated booth, satisfying established standards (ANSI S3.1–1999). Thresholds were measured for octave frequencies from 125 to 8000 Hz with GSI 61 (Grason-Stadler, Madison, WI, USA) systems calibrated according to recommended criteria (ANSI S3.6–1996). Threshold values at each frequency were obtained to within 5 dB via air conduction or bone conduction, according to modified Hughson-Westlake procedure (ANSI S3.21–1978 (R-1992)). Because the time of the first hearing test was different according to the accompanying injuries, pure tone thresholds measured at follow-up visits rather than initial assessment was used to detect the presence of hearing impairment. The average bone conduction thresholds at 0.5, 1, 2, and 4 kHz were calculated for each ear and used to stratify the severity of SNHL. Given their low incidence, the severity of hearing impairment was simply classified into three groups as follows: normal (≤25 dB HL), mild to moderate (26–60 dB HL), and severe to profound (>60 dB HL). In the cases of hearing loss already known before trauma, they were included in the normal group when the difference compared to data prior to the trauma or the difference between 2 ears was less than 10 dB HL.

### Videonystagmography

The slow phase velocities of spontaneous nystagmus and head-shake nystagmus were measured using a VNG system (I-Portal, Neuro Kinetics Inc, Pittsburgh, PA, USA) with the subject in the sitting position. HSN was assessed after 15 seconds of passive head shaking at a frequency of 2 Hz with 30° neck flexion. After subtracting the amount of SN, the intensity and direction of the horizontal component of corrected HSN were obtained as parameters for analysis. The criteria of HSN were defined as having been met when the following values were exceeded: horizontal 3° per second; vertical 2° per second; torsional 2° per second, and when the nystagmus lasted more than 5 seconds.

### Bithermal caloric test

The bithermal caloric test was performed in the supine position with upward neck flexion of 30°. Eye movements were recorded using a binocular video oculography system (I-Portal, Neuro Kinetics Inc., Pittsburgh, PA, USA) to track horizontal eye movements. Caloric irrigation was performed using binaural alternate irrigation for 30 seconds with 300 mL of cold (30°C) and warm (44°C) water (ICS NCI-480, GN Otometrics, Taastrup, Denmark). The interval between individual irrigations was at least 5 minutes. The result was considered abnormal when the unilateral weakness was over 25%.

### Statistical analysis

Association between the degree of TBP and the prevalence of otic-capsule violation, SNHL, FNP, vestibular dysfunction, and BPPV were evaluated by the linear-by-linear association test. Fisher’s exact test was used to compare the VFT results by the presence of otic-capsule violation because of the small sample size. Statistical significance was set at P < 0.05. All statistical analyses were performed with the aid of SPSS software (version 18.0; SPSS, Inc., Chicago, IL, USA).

## Results

A total of 49 patients were included in this study. The mean age was 47.9 years (22–74), and most patients were male (n = 41, 83.7%). Falls were the most common mechanism of injury, present in 53.1% (n = 26) of cases. It is notable that motor vehicle accidents (MVAs) accounted for 36.7% (n = 18) of cases. Among 18 cases of MVA, 7 were motorcycle riders, 10 were pedestrians, and only 1 patient was a car passenger. Blunt assaults and heavy object falling were responsible for 4.1% (n = 2) and 6.1% (n = 3) of cases respectively.

The distribution was fracture was unilateral in 44 patients and bilateral in 5 patients. Of the 44 patients with unilateral TBFs, the right temporal bone was affected in 25 and the left in 19. Of the 54 fractures reviewed, 6 (11.1%) were comminuted fractures that had multiple fracture lines. According to the traditional classification system, 9 (18.8%) had transverse fractures, 18 (37.5%) had longitudinal fractures, and 21 (43.8%) had oblique fractures among TBFs with single fracture component. According to the new system, 49 (90.7%) had OCS fractures, and 5 (9.3%) had OCV fractures.

**Fig 3:**
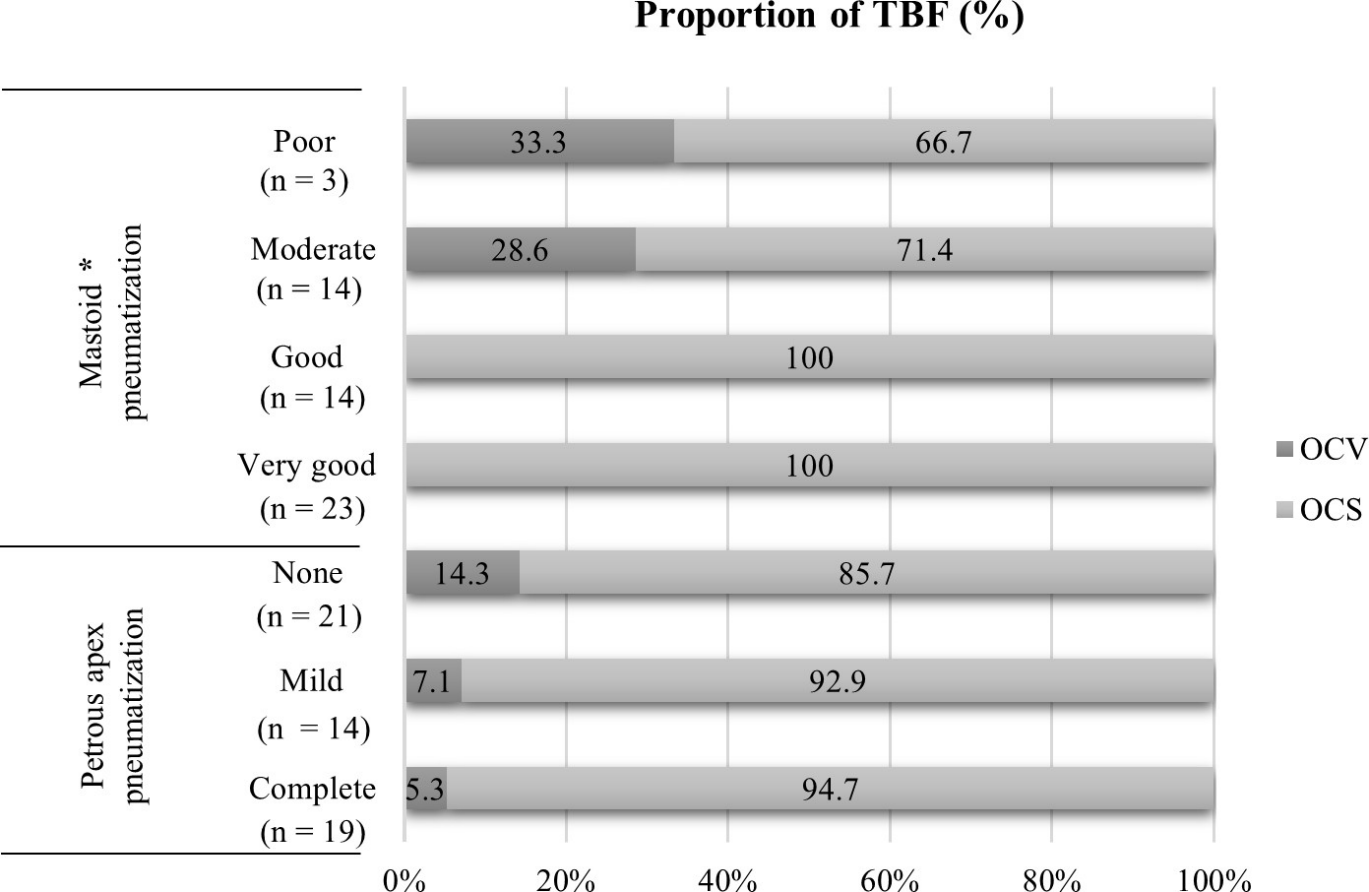
Proportion of temporal bone fractures with otic-capsule violation. Temporal bones with the reduced pneumatization were more likely to have OCV fractures than those with good pneumatization. TBF, temporal bone fracture; OCV, otic-capsule violating; OCS, otic-capsule sparing; asterisk, statistically significant (P< 0.05).

The proportion of OCV fractures stratified by the pneumatization pattern of the mastoid and petrous apex is presented in Fig 3. The mastoid pneumatization classified with reference to the sigmoid sinus did demonstrate statistically significant correlation with the violation of the otic-capsule bone (P = 0.002). The otic-capsule was not violated in 100% of the temporal bones with “good” and “very good” mastoid pneumatization. Although the percentage of OCV fractures was progressively lower with increasing pneumatization of the petrous apex, which did not reach statistical significance (P = 0.326).

**Fig 4:**
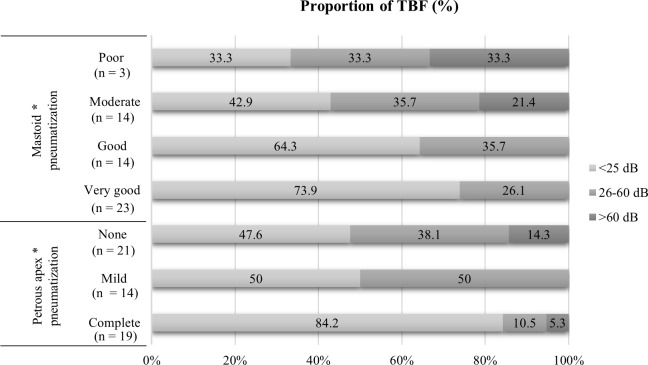
Proportion of temporal bone fractures with sensorineural hearing loss (SNHL). The less pneumatized temporal bone tended to have the greater the possibility of SNHL and the more severe degree of SNHL. The severity of SNHL was stratified using the average bone conduction thresholds at 0.5, 1, 2, and 4 kHz. TBF, temporal bone fracture; asterisk, statistically significant (P< 0.05).

Clinical sequalae of temporal bone trauma stratified by the degree of pneumatization in the mastoid and petrous apex are summarized in Table 1. Of the 54 TBFs, 33 (61.1%) had normal hearing or temporary hearing loss recovered completely, and 21 (38.9%) had persistent SNHL at the most recent follow-up assessment. Two subjects already had mild SNHL before trauma, diagnosed as presbycusis and noise induced hearing loss, respectively. Because the impairment of hearing was less than 10 dB HL after trauma, they were included in the normal group. The severity of SNHL were mild to moderate in 17 cases, and severe to profound in 4 cases. There were significant associations between the severity of SNHL and the degree of pneumatization in both entire temporal bone and petrous apex region (P = 0.005 and 0.024, respectively). These results are detailed in Fig 4. Fig 4 shows that the less pneumatization of the temporal bone tended to have the greater the possibility of SNHL and the more severe degree of SNHL.

**Table 1:** Complication rate by the mastoid and petrous apex pneumatization degree.

Pneumatization degree		Number of cases (%)			
		SNHL	FNP	Vestibulopathy	BPPV
Mastoid^**a**^	Poor (n = 3)	2 (66.7)	1 (33.3)	1 (33.3)	1 (33.3)
	Moderate (n = 14)	8 (57.1)	3 (21.4)	4 (28.6)	2 (14.3)
	Good (n = 14)	5 (35.7)	3 (21.4)	0/13 (0)	3/13 (23.1)
	Very good (n = 23)	6 (26.1)	6 (26.1)	5/22 (22.7)	2/22 (9.1)
P-value		0.037	0.927	0.615	0.360
Petrous apex^b^	None (n = 21)	11 (52.4)	7 (33.3)	3 (14.3)	4 (19.0)
	Mild (n = 14)	7 (50.0)	1 (7.1)	5 (35.7)	2 (14.3)
	Complete (n = 19)	3 (15.8)	5 (26.3)	2/17 (11.8)	2/17 (11.8)
P-value		0.020	0.578	0.925	0.536
Total		21/54 (38.9)	13/54 (24.1)	10/52 (19.2)	8/52 (15.4)

SNHL, sensorineural hearing loss; FNP, facial nerve palsy; BPPV, benign paroxysmal positional vertigo.

^a^The degree of mastoid pneumatization according to Han’s classification system [16].

^b^The degree of petrous apex pneumatization according to Jadhav’s classification system [17].

P-values were calculated using the linear-by-linear association test.

Within 14 days after temporal bone trauma, the presence of FNP was noted in 13 (24.1%) TBFs, of which 8 had noted to have delayed FNP. Four patients underwent facial nerve decompression. With both classifications of the pneumatization of the temporal bone, there was no significant difference of prevalence rate of FNP (Table 1).

FNP was associated with 1 of 9 (11.1%) transverse fractures, 5 of 18 (27.8%) longitudinal fractures, 3 of 21 (14.3%) oblique fractures, and 4 of 6 (66.7%) comminuted fractures. The differences in rates of FNP between fractures classified according to the traditional system were not significant. However, FNP was significantly increased in comminuted fractures (P = 0.025). TBFs with multiple components (4/6, 66.7%) were 3.5 times more likely to have accompanying FNP than those with a single component (9/48, 18.8%).

Of the 48 patients (52 TBFs) who had adequate documentation to examine dizziness after trauma, 18 patients (37.5%) reported dizziness. Vestibular function test (VFT) was performed when patients had symptoms and/or examination findings that were suggestive of peripheral vestibular disorders. Of the 18 patients who underwent VFT, 10 patients had vestibular dysfunction, and 8 were diagnosed with BPPV. We also compared the incidence of VFT results for each classification systems of TBP. The prevalence of vestibular dysfunction and BPPV tended to be higher in the reduced pneumatization group, although this finding was not statistically significant (Table 1). When classified according to the presence of or absence of otic-capsule violation, the incidence of vestibular dysfunction was 60% in OCV fractures and 14.9% in OCS fractures. The difference in the prevalence of vestibular dysfunction was statistically significant (P = 0.043).

## Discussion

To our knowledge, this is the first study to report a direct correlation between the degree of TBP and the severity of clinical sequelae associated with TBF in a large group of adult patients. The patients are predominantly male (83.7%), and injury by falls (53.1%) is the most common. Our findings indicate that the degree of TBP showed a significant association with functional deficits from injury to structures within temporal bone. OCV fractures were present only in temporal bones with reduced pneumatization. In OCV fractures, persistent SNHL, including 40% with mild to moderate (26–60 dB) and 60% with severe to profound (>60 dB), and vestibular dysfunction (60%) were more frequently found. The greater the degree of pneumatization tended to have the less the possibility of SNHL and the less the degree of SNHL. However, facial nerve injury after TBF was less likely to have correlation with the degree of TBP. This is likely the result of a long, complex, and tortuous intraosseous course of the facial nerve, given the potential for injury in any part of the pathway from high energy trauma. Our results supported the hypothesis that TBP acts as a shock absorber to protect surrounding vital structures in trauma.

In the current study, the degree of TBP was measured using the validated classification systems proposed by Han et al [16] and Jadhav et al [17]. Although air volume measured on 3D reconstruction is theoretically more accurate, it is not a practical technique in subjects with TBF [18]. In contrast, the sigmoid sinus and the carotid canal are consistently identifiable reference structure on the axial view of TBCT, which can be applied to the fractured temporal bone with good feasibility, and analysis using these structures was found to significantly represent the degree of pneumatization in the entire temporal bone and the petrous apex, respectively. Although the association between the pneumatization groupings for the 2 reference structures was significant, the numbers of cases in each group were different. We investigated whether the compartment of each of TBP allows for protection of different vital structures from head trauma. Only mastoid pneumatization showed significant protective effect for the otic-capsule itself, while patterns of clinical sequelae were not different from those of the petrous apex.

TBFs are associated with various types of hearing loss, including conductive, sensorineural, or mixed. Post-traumatic hearing loss may be transient with spontaneous resolution or permanent. We focused on permanent hearing loss that persists after a period of recovery subsequent to the trauma. Therefore, transient hearing loss that recover to baseline levels in the weeks following trauma and persistent conductive hearing loss with normal bone conduction thresholds which usually can be corrected surgically were not considered in this study. Our study found that 38.9% of TBFs presented with persistent SNHL, either pure SNHL or mixed hearing loss. OCV fractures which lead to disruption of the membranous labyrinth were associated with a 100% incidence of SNHL as expected. Even without damage to the otic capsule, SNHL could occur due to avulsion or trauma to the cochlear nerve, interruption of the cochlear blood supply, hemorrhage in the cochlea, perilymphatic fistulas or obstruction of the endolymphatic duct [19]. In the literature, microfractures involving the otic capsule which would not be visible on CT scan were identified by histopathologic analysis [20]. Accordingly, persistent SNHL also occurred in 32.6% of our cases without OCV fractures on TBCT. According to shock absorption spaces theory, the less the degree of TBP, the greater the damage of the cochlea and the greater the degree of SNHL. Our results showed that the degree of TBP of the patients with or without SNHL have statistical difference. As for the severity of hearing impairment, our cases with reduced TBP presented a significantly severer hearing loss than those with increased TBP. Mun et al. hypothesized that the distance between the otic capsule and fracture line would be correlated with the degree of damage to the cochlea in OCS fractures [21]. Although the shortest length between the otic capsule and fracture line on TBCT showed a significant association with degree of hearing impairment in patients with SNHL, their study had some limitations. Regardless of the course in the three-dimensional perspective, the distance was measured only on axial section in terms of fracture lines rather than fracture planes. As a result, the measuring points on the otic-capsule were not constant varied from the horizontal semicircular canal to the basal turn of the cochlea. Moreover, the degree of TBP was not considered. Turgut and Tos found that the length of mastoid process was significantly longer in temporal bones with large pneumatization than those with small [22]. Thus, we think the temporal bone in patients with SNHL tend to have reduced pneumatization, which results in the shorter length between the otic capsule and the fracture line being measured.

The overall incidence rate of FNP in our TBF cases was 24.1%, which falls within published rates of 7% to 23% [23, 24]. Interestingly, the incidence of FNP was significantly higher in cases of comminuted fracture. Since higher energy is required to fragment the temporal bone, it is possible that the greater energy of impact would be delivered to induce the fractures associated with FNP. However, it is not possible to directly compare actual impact forces in the clinical situation. Thus, we investigated the prevalence and pattern of the associated intracranial hemorrhage (ICH) which can be indicative of high trauma forces involved in head trauma [25]. We found the high incidence of ICH (40/49, 81.6%) among patients with TBF, similar to the findings of previous studies (78–84%) [25–27]. Subdural hematoma (n = 19, 38.8%) was the most common type, followed by subarachnoid hemorrhage in 34.7% (n = 17), and epidural hematoma in 10.2% (n = 5). In our study, all 6 patients with comminuted fractures were diagnosed to have ICH, and the majority of FNP patients (11/13, 84.6%) also had some kind of ICH. Although, we did not find any significant association between any types of ICH and the injury of the facial nerve, this would suggest that more severe forces were delivered in cases of FNP. Our results show no significant association between the degree of TBP and FNP. This finding does not seem to contradict the theory that TBP absorbs and disperses the impact energy. Regarding the mechanism of the traumatic facial nerve injury, sharing forces produced by sudden acceleration/deceleration result in intraneural contusion, edema, hemorrhaging, and consequent ischemia [28]. Although TBP may provide a distance buffer from an initial impact allowing for deceleration, high deceleration from high energy impacts would lead to high chances of FNP. Alternatively, the degree of TBP may be irrelevant if the force of impact is severe enough to fracture the otic capsule known as one of the densest bones in the body or result in a comminuted fracture of the temporal bone. Because severe TBFs with otic capsule violation and/or comminution often result in severe brain injury, the incidence of FNP may also have been underestimated as these patients frequently do not survive their head injuries or have significant cognitive deficits if they do survive.

This study was limited by its retrospective design. The clinical data available during the initial acute management period were limited for many patients in this study. From high energy trauma such as falls and motor vehicle accidents, TBF rarely occurred in isolation and other comorbidities were frequent in patients with TBF [27]. Most patients were initially managed by a neurosurgeon or orthopedic surgeon, and then referred to the otologist later. Furthermore, situations such as altered mental status, or the use of sedative agents often lead to the delay in otologic evaluation. This may lead to a biased result, especially for post-traumatic dizziness. The vertigo after temporal bone trauma is known to be usually self-limiting and resolve with central vestibular compensation and partial restoration of peripheral labyrinthine function [29]. In a series of 113 temporal bone fractures, 14.4% of patients reported dizziness which resolved spontaneously prior to discharge [26]. In another series, 91.7% of patients who had OCV fractures exhibited a subjective improvement of dizziness after a couple of weeks [30]. In the present study, VFTs were not performed on all patients and the diagnosis of dizziness was primarily made by the patient’s self-report. Therefore, symptom-free vestibulopathy at the time of evaluation could be very difficult to differentiate from truly unaffected condition. The incidence of vestibular dysfunction reported in the present study may be an underestimation: 60% in OCV fractures and 14.9% in OCS fractures. Choi et al. reported a 100% incidence of vestibular dysfunction in 10 cases of OCV fractures [30]. Based on this data, two OCV fracture patients in our series who did not complain of subjective dizziness may have vestibular dysfunction. Our incidence of vestibular dysfunction in OCS fractures was also lower than the reported incidence of 30.6% in patients with dizziness after MVA, even though they excluded patients showing a definite facture of the temporal bone or the presence of intracranial lesion [31]. However, our incidence of traumatic BPPV (16.7%) is within the rage of 16–29% incidence quoted in the recent publication [32]. Traumatic BPPV usually develops days to weeks after injury [29]. We assume that the incidence of traumatic BPPV was not affected by the delay of evaluation by an otologist. When focusing on the clinical sequelae of TBF, it may be that the actual vestibular dysfunction is a less important factor than is the presence of prolonged symptoms, including vertigo, imbalance, and disequilibrium. The persistent traumatic vertigo may require additional interventions to resolve, such as vestibular rehabilitation therapy, repositioning maneuvers for BPPV, or even surgical treatment for perilymphatic fistula. It also appears that patients with persistent traumatic vertigo are far less likely to return to work [33].

## Conclusions

In this study, the correlation between the degree of pneumatization and clinical sequalae from temporal bone fracture was investigated. The degree of pneumatization in the temporal bone was negatively correlated to the incidence of OCV fracture and the severity of hearing impairment in patients with temporal bone fracture. This study substantiated the potential protective effect of temporal bone pneumatization in TBFs.
